# Type 2 Diabetes Restricts Multipotency of Mesenchymal Stem Cells and Impairs Their Capacity to Augment Postischemic Neovascularization in *db/db* Mice

**DOI:** 10.1161/JAHA.112.002238

**Published:** 2012-12-19

**Authors:** Jinglian Yan, Guodong Tie, Shouying Wang, Katharine E. Messina, Sebastian DiDato, Sujuan Guo, Louis M. Messina

**Affiliations:** Division of Vascular and Endovascular Surgery, University of Massachusetts Medical School, Worcester, MA

**Keywords:** limb ischemia, mesenchymal stem cells, Nox4, oxidant stress, type 2 diabetes

## Abstract

**Background:**

This study tested the hypothesis that type 2 diabetes restricts multipotency of *db/db* mesenchymal stem cells (MSCs), promotes their terminal differentiation into adipocytes rather than endothelial cells, thereby promotes adipocytic infiltration into ischemic muscles, and reduces their capacity to participate in postischemic neovascularization.

**Methods and Results:**

To test this hypothesis, we transplanted MSCs from *db/db* or wild-type (WT) mice into WT recipients after induction of hind limb ischemia. WT recipients of *db/db* MSCs demonstrated adipocyte infiltration of ischemic muscle and impaired neovascularization; WT recipients of WT MSCs showed no intramuscular adipocyte infiltration and had significantly enhanced neovascularization (*P*<0.05; n=6). Confocal microscopy showed that the percentage of MSCs that differentiated into an adipocyte phenotype was greater and into an endothelial cell was less in WT recipients transplanted with *db/db* MSCs than those transplanted with WT MSCs (*P*<0.05; n=6). In vitro, *db/db* MSCs exhibited greater oxidant stress, greater adipocyte differentiation, and less endothelial differentiation than WT MSCs, and these differences were reversed by treatment with *N*-acetylcysteine or Nox4 siRNA (*P*<0.05; n=6). Insulin increased Nox4 expression, oxidant stress, and adipocyte differentiation in WT MSCs, and these insulin-induced effects were reversed by Nox4 siRNA (*P*<0.05; n=6). Reversal of *db/db* MSC oxidant stress by in vivo pretreatment with Nox4 siRNA before transplantation reversed their impaired capacity to augment postischemic neovascularization.

**Conclusions:**

Type 2 diabetes–induced oxidant stress restricts the multipotency of MSCs and impairs their capacity to increase blood flow recovery after the induction of hind-limb ischemia. Reversal of MSC oxidant stress might permit greater leverage of the therapeutic potential of MSC transplantation in the setting of diabetes.

## Introduction

Type 2 diabetes mellitus is a major risk factor for the development of vascular disease, including symptomatic peripheral artery disease.^1^ This association reflects the increased rate of atherosclerosis in these patients^2^; however, it also reflects a reduced capacity for postischemic neovascularization in patients who have type 2 diabetes.^3,4^ The orchestration of events that make up postischemic neovascularization is incompletely understood, although it is known that bone marrow–derived cells, including mesenchymal stem cells (MSCs), participate in this process.^5–7^ MSCs are a heterogeneous stromal cell population that have the capacity to self-renew as well as to differentiate into a variety of terminally differentiated cells, including adipocytes, chondrocytes, endothelial cells, and osteocytes.^8–10^ MSCs also have the capacity to home in on sites of vascular injury, to engraft into damaged blood vessels, to differentiate into vascular cells, and to exert a paracrine effect by the local release of vascular growth factors and cytokines.^5,7,9^ Although we know that type 2 diabetes impairs neovascularization, the extent to which this impaired neovascularization is a result of the effects of type 2 diabetes on MSC function is unknown.

We have shown previously that postischemic neovascularization is significantly impaired in type 2 diabetic *db/db* mice. In this study, *db/db* mice also exhibited substantial adipocyte infiltration within the ischemic hind-limb muscles during ischemic neovascularization, a finding not present in type 1 diabetic mice or in wild-type (WT) mice.^4,11^ The established capacity of MSCs for adipocytic differentiation led us to hypothesize that the adipocytes in postischemic muscle in *db/db* mice are derived from MSCs. It has also been shown that Nox4-induced reactive oxygen species (ROS) are essential for the terminal differentiation of rat bone marrow–derived mesenchymal stem cells into adipocytes.^12^ We therefore hypothesized that type 2 diabetes-induced Nox4 expression restricts *db/db* multipotency of MSCs in a manner that promotes their terminal differentiation into adipocytes rather than endothelial cells and thereby reduces their capacity to augment postischemic neovascularization.

To test these hypotheses, bone marrow–derived MSCs were harvested from *db/db* and WT mice, were transduced with an adenovirus expressing green fluorescent protein (GFP), and then were transplanted into WT recipient mice 24 hours after induction of hind-limb ischemia. WT recipient mice receiving *db/db* MSCs exhibited less foot blood flow recovery than did recipient mice receiving WT MSCs and showed extensive adipocytic infiltration within the ischemic gastrocnemius muscle. GFP-labeled *db/db* MSCs, but not WT MSCs, colocalized with perilipin in ischemic muscle, indicating that the adipocytes were derived from transplanted MSCs. Corresponding in vitro and in vivo experiments showed that hyperinsulinemia-induced Nox4 activity generated the oxidant stress that restricted MSC multipotency and impaired the capacity of MSCs to augment postischemic neovascularization.

## Methods

### Animals

Male C57BL/6 and B6.Cg-*m* +/+ Lepr^db^/J mice were purchased from Jackson Laboratories (Bar Harbor, ME). Unless otherwise specified, mice were fed ad libitum using a standard chow (Teklad #7012) that contains 5.7% fat by weight. Diet-induced diabetes was generated by feeding C57BL/6 mice a high-fat diet for 2 months (D12492 from Research Diets; 34.9% fat by weight).^13^ All protocols were approved by the Institutional Care and Use Committee of the University of Massachusetts Medical School.

### Antibodies and Reagents

Reagents were obtained as follows: MesenCult Basal Medium and Adipogenic Stimulatory Supplements (mouse) from Stem Cell Technologies (Vancouver, BC); VEGF, StemXVivo Base Medium, and Osteogenic Supplements from R&D Systems (Minneapolis, MN); *N*-acetylcysteine, insulin, and 2,7-dichlorofluorescein diacetate from Sigma-Aldrich (St. Louis, MO); Lipofectamine RNAiMax from Invitrogen (Carlsbad, CA); and Oil Red O from Poly Scientific (Bay Shore, NY). Antibodies were obtained as follows: CD31 and nitrotyrosine from BD Bioscience (San Diego, CA); adiponectin and PPARγ from Sigma-Aldrich; Nox1, Nox2, and Nox4 from Abcam (Cambridge, MA); eNOS and Ser^1188^ phosphorylated eNOS from Cell Signaling (Danvers, MA); perilipin from Fitzgerald Industries International (Acton, MA); and Cy3-conjugated goat anti-rat secondary antibody from Jackson Immunoresearch (West Grove, PA).

### Isolation and Expansion of MSCs

**Figure 1.**
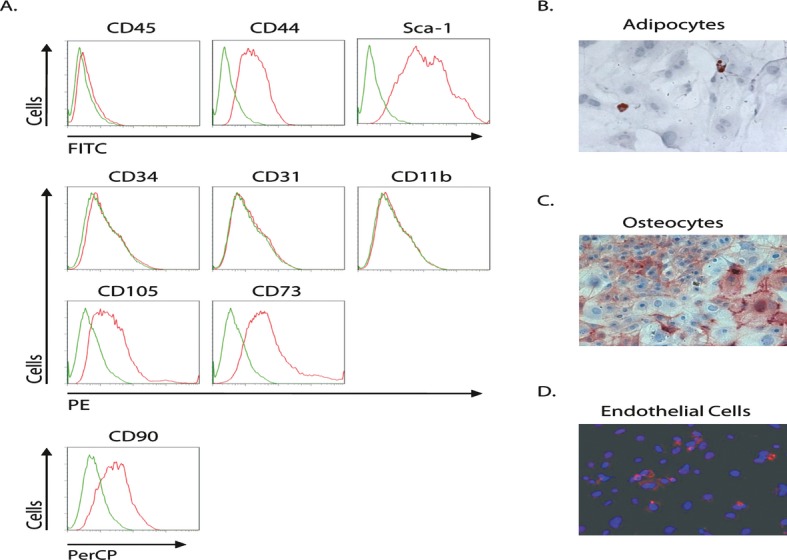
Identification of MSCs. A, Passage 3 MSCs from WT mice demonstrated expression of CD44 (79%), CD73 (89%), CD105 (82%), and Sca-1 (91%), but not CD31, CD34, CD11b, or CD45. In all histograms, the red line represents the cells stained with specific antibody, whereas the green line represents the isotype-matched control IgG B through D, Passage 3 MSCs from WT mice demonstrated differentiation to adipocytes, osteocytes, and endothelial cells in response to selective differentiation media. MSCs indicates mesenchymal stem cells; WT, wild-type.

Femurs and tibias were harvested, and the bone marrow was flushed with sterile PBS. Bone marrow mononuclear cells were plated in untreated plastic flasks using MesenCult Basal Medium. After 3 days, the nonadherent cells were removed by washing with PBS. The remaining cells were grown until 70% confluent, whereupon cells were passaged. All cells used in these experiments were in passages 3 to 4 (P3-4). The identity of passage 3 cells was confirmed using established criteria of the MSC phenotype. First, cells expressed CD44, CD73, CD105, and Sca-1, but did not express CD11b, CD31, CD34, or CD45 (Figure 1A). Second, the cells could be differentiated into mesodermal (adipocytes, osteocytes) and nonmesodermal (endothelial cells) lineages by in vitro exposure-selective differentiation media (Figure 1B through 1D).^7,13^

To evaluate the effects of hyperinsulinemia on mesenchymal stem cells, P3-4 mesenchymal stem cells were treated with different concentration of insulin (10, 100, 174, 348, or 1740 nmol/L) for 2 days, and then MSC differentiation capacity was analyzed. To evaluate the effects of hyperglycemia, P3-4 mesenchymal stem cells were treated with different concentrations of glucose (5.6, 10, 20, or 30 mmol/L) for 2 days, and then MSC differentiation capacity was analyzed.

### Western Blotting

MSCs were homogenized (50 mmol/L HEPES [pH 7.5], 150 mmol/L MgCl_2_, 1 mmol/L EDTA, 100 mmol/L NaF, 1% NP40). Protein extracts were subjected to SDS-PAGE, transferred to nitrocellulose membranes, and probed for nitrotyrosine, eNOS, P-eNOS, adiponectin, PPARγ, or Nox1, Nox2, and Nox4.

### Colony-Forming Unit Assay

Bone marrow mononuclear cells (1×10^4^) were seeded on 24-well plates in mesenchymal culture medium α-MEM with supplementation of 20% FCS for 14 days. Cells were stained with 0.1% Toluidine blue (Sigma-Aldrich, St. Louis, MO) in 1% paraformaldehyde; stained aggregates >50 cells were scored as CFU-F colonies under a Leica stereomicroscope.

### Hind-Limb Ischemia and Flow Recovery Measurement

Unilateral hind-limb ischemia was generated by femoral artery ligation and excision under 2% isoflurane anesthesia.^4^ A laser Doppler perfusion imager (Moor Instruments Ltd., Devon, UK) was used to simultaneously measure blood flow to both hind-limb feet. Blood flow to the ischemic foot was expressed as a percentage of flow to the nonischemic foot.

### MSC Differentiation Assays

MSCs were seeded in 8-well chamber slides. After 24 hours, the medium was changed to a selective differentiation medium, as follows: for adipocytes, MesenCult Basal Medium with Adipogenic Stimulatory Supplements (mouse); for endothelial cells, MesenCult Basal Medium with 50 ng/mL VEGF; for osteocytes, StemXVivo Base Medium with Osteogenic Supplements. After 72 hours, cells were stained with Oil Red O to identify adipocytes, with CD31- and Cy3-conjugated secondary antibody to identify endothelial cells, or with Fast Violet B, napthol, and Mayer's hematoxylin to identify osteocytes. A blinded observer counted ≥50 randomly selected fields, and data were expressed as the number of specifically stained cells divided by total cells in the field at ×200 magnification. This percentage was averaged and used as a single data point for each MSC experiment.

### MSC Transplantation

**Figure 2.**
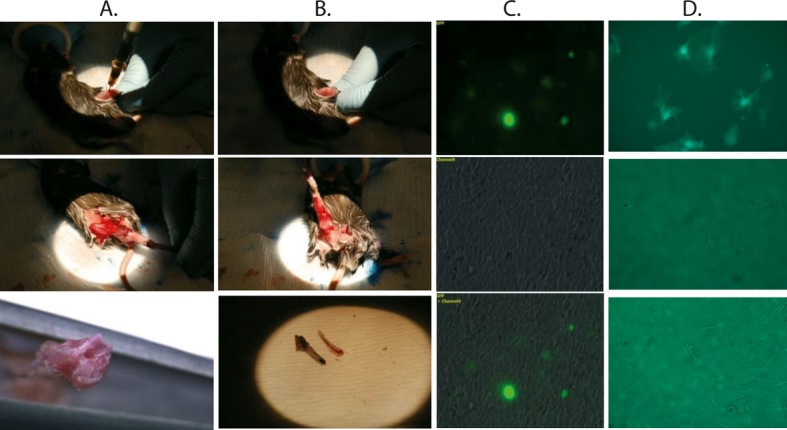
MSC intrabone marrow transplantation. A and B, Intrabone marrow transplantation. C, Bone marrow smears after 14 days of GFP-labeled MSC intrabone marrow transplantation. D, In vitro culture of Ad-GFP-transfected MSCs after 14 days. MSC indicates mesenchymal stem cell; GFL, green fluorescent protein.

MSCs harvested from *db/db* or WT mice were used in MSC transplantation studies. All transplantation recipients were WT mice that underwent unilateral femoral artery excision to induce hind-limb ischemia. One day later, 10^6^ MSCs suspended in 10 μL of sterile PBS were transplanted into the femoral bone marrow cavity in the ischemic hind limb (Figure 2A and 2B). To this end, the knee was flexed to 90°, and a microsyringe needle was inserted through the patellar tendon of the ischemic hind limb into the marrow cavity.^14^ In some studies, MSCs were transduced with a replication-deficient adenovirus conjugated to GFP 24 hrs prior to transplant. The transduction efficiency was 90%, and the percentage of MSCs that expressed GFP in vitro did not change over 14 days (Figure 2C and 2D). We have previously shown that intravenous infusion of an empty adenovirus vector into the ischemic hind limb does not cause any change in postischemic neovascularization or any evidence of increased inflammatory response in the ischemic hind limb.^15^

### Muscle Microscopy

The gastrocnemius muscle was removed from the ischemic hind limb, embedded in OCT, frozen on dry ice, and stored at −80°C. Ten micromolar cryosections were stained with Oil Red O and counterstained with hematoxylin to determine intermuscular adipocyte infiltration. Colocalization studies of GFP with perilipin or of GFP with CD31 were carried out to determine if transplanted MSCs differentiated into adipocytes or endothelial cells, respectively. Images were obtained with a Zeiss Imager M1 microscope and AxioVision 4.6 software or by confocal microscopy using a Solamere Technology Group CSU10B Spinning Disk Confocal Microscope (Digital Light Microscopy Core, University of Massachusetts Medical School). Image Z-series were taken with a ×40 objective using a Z-step size of 0.20 μ. The percentage of colocalization was determined in at least 10 randomly selected fields by a blinded observer and expressed as a percentage of total GFP-positive cells in the field. This percentage was averaged and used as a single data point for each MSC experiment.

### Nox4 siRNA Knockdown Studies

Nox4 siRNA sequences were as follows^16^: sense, 5′-GACCUGACUUUGUGA-ACAUTT-3′; antisense, 5′-AUGUUCACAAAAUCAGGUCTT-3′. siRNA experiments were controlled using Cy3-conjugated nonsense siRNA. All RNA was obtained from Applied Biosystems/Ambion (Austin, TX). Transfection was carried out using Lipofectamine RNAiMAX. The efficiency of transfection was ≍90%, as determined by immunofluorescent microscopic inspection of cells for Cy3.

### Tubular Formation Assay

Passage 3-4 MSCs from *db/db* or WT mice were cultured in angiogenic medium for 3 days and then seeded on BD Matrigel Matrix on 24-well plates and incubated at 37°C in a 5% CO_2_ atmosphere. Tubular images were taken with an Olympus inverted microscope after 24, 48, and 72 hours.

### Transplantation of siNox4-Transfected db/db MSCs

To determine if pretreatment of *db/db* MSCs with Nox4 siRNA could restore their differentiation capacity in vivo and rescue their impaired capacity to augment postischemic neovascularization in WT mice, we transplanted MSCs from WT, *db/db* MSCs or siNox4-transfected *db/db* MSCs into WT recipients 24 hours after induction of hind-limb ischemia. Blood flow recovery measurement was done by laser Doppler perfusion imaging (LDPI) at different times after induction of hind-limb ischemia.

### Immunostaining for Collateral Diameter and Capillary Density

Ischemic gastrocnemius and thigh muscles were collected 28 days after induction of hind-limb ischemia. Capillary density was quantified by CD31 immunostaining on 10-μm-thick frozen sections of ischemic gastrocnemius muscle. Capillary density was determined as the mean capillary/myofiber ratio. Collateral artery diameter was determined by counting the number of CD31- and α-SMA-positive arteries in thigh muscles from the ischemic limb on 10-μm-thick frozen sections. Collateral artery diameter was measured using precalibrated microscope calipers (Carl Zeiss, Gottingen, Germany) in a blinded manner.

### Statistical Analysis

Results are expressed as mean±standard error of the mean (SEM). Data were evaluated for normality using a Kolmogorov**–**Smirnov test along with visual inspection of histograms of unstandardized residuals. Data that did not deviate from a Gaussian distribution were analyzed using parametric tests; otherwise, nonparametric tests were used. Student *t* tests and Mann–Whitney *U* tests were used as appropriate when comparing 2 groups. When comparing >2 groups, 1-way ANOVA or Kruskal–Wallis tests were used along with post hoc comparisons (Tukey's and Dunn's tests, respectively) where appropriate. For repeated-measures data, either a 2-way repeated-measures ANOVA was used with Bonferroni post hoc tests or a mixed-model ANOVA was used with Tukey post hoc comparisons. For count data from colony-forming experiments, Poisson regression analysis was used.

All statistical analyses were performed using SPSS version 19.0.0 (SPSS, Inc, Chicago, IL; http://www.spss.com), GraphPad Prism version 5.04 (GraphPad Software, San Diego, CA; http://www.graphpad.com), or SAS version 9.2 (SAS Institute Inc, Cary, NC) software. Group differences with *P*<0.05 were deemed statistically significant.

## Results

### Transplantation of Bone Marrow–Derived *db/db* MSCs Causes Adipocyte Infiltration of Ischemic Hind-Limb Muscles and Impairs Postischemic Foot Blood Flow Recovery in WT Recipient Mice

**Figure 3.**
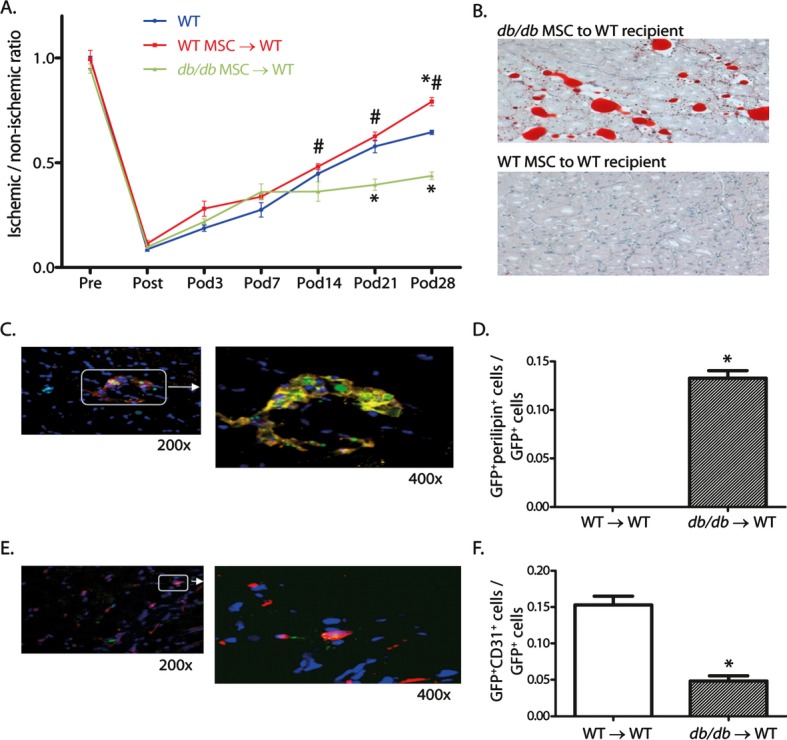
Foot blood flow recovery, muscle histology, and colocalization studies in WT mice transplanted with *db/db* or WT MSCs after induction of hind-limb ischemia. A, Foot blood flow recovery by LDPI (mean±SEM, n=6; *P<0.05 vs WT, #*P*<0.05 vs *db/db* MSC→WT transplant group). B, Histology of gastrocnemius muscle from the ischemic hind limb (Oil Red O for identification of adipocytes, hematoxylin counter stain, ×200). C, Representative confocal images (GFP for identification of MSCs [green] and perilipin for identification of adipocytes [red]). D, Ratio of GFP^+^periplipin^+^ cells to GFP^+^ cells in the ischemic hind-limb muscle (mean±SEM, n=5; *P<0.05). E, Representative confocal images (GFP for identification of MSCs [green] and CD31 for identification of endothelial cells [red]). F, Ratio of GFP^+^CD31^+^ cells to GFP^+^ cells in the ischemic hind-limb muscle (mean±SEM, n=5; *P<0.05). WT indicates wild-type; MSCs, mesenchymal stem cells; LDPI, laser Doppler perfusion imaging; and GFL, green fluorescent protein.

We first compared foot blood flow recovery of recipient WT mice with that of nontransplanted WT C57BL/6 mice (Figure 3A). In WT mice, blood flow in the foot of the ischemic limb on postoperative day 28 recovered to 64±1% of that in the nonischemic limb. After MSC transplantation, we found that in WT recipients transplanted with WT MSCs, blood flow in the foot of the ischemic limb increased to 79±2% of that in nonischemic limb, significantly higher than that in WT mice without MSC transplantation. Thus, a therapeutic effect of MSC transplantation was noted. In contrast, WT recipients of *db/db* MSCs recovered foot blood flow to only 44±2% of that in the nonischemic limb, significantly less than in WT mice and in WT mice that received WT MSCs. Thus, transplantation of *db/db* MSCs into WT mice decreased limb blood flow recovery below that found in nontransplanted C57BL/6 mice.

Two weeks after induction of ischemia, we observed adipocytic infiltration of the ischemic gastrocnemius muscle of WT recipient mice that received *db/db* MSCs (Figure 3B). Many areas of clustered adipocytes as well as single adipocytes were spaced irregularly between muscle fibers within the body of the gastrocnemius muscle in a pattern identical to that previously reported in *db/db* mice.^4^ Adipocytes were not observed in the ischemic gastrocnemius muscle of recipient mice receiving WT MSCs.

### *db/db* MSCs Transplanted Into WT Recipients Differentiate More Frequently Into an Adipocyte Rather Than Into an Endothelial Cell Phenotype Within Ischemic Gastrocnemius Muscle

We next sought to confirm that the adipocytes within the ischemic muscle of WT recipients of *db/db* MSCs are derived from the transplanted MSCs. Two weeks after induction of hind-limb ischemia, immunofluorescent and confocal microscopy showed colocalization of GFP and perilipin within ischemic muscle in WT recipients that received *db/db* MSCs. This colocalization was not observed in WT recipient mice receiving WT MSCs (Figure 3C and 3D). The opposite pattern was noted when colocalization of GFP and CD31 within the ischemic gastrocnemius muscle was measured. Recipients of WT MSCs had more cells that showed GFP–CD31 colocalization within ischemic muscle than did recipients of *db/db* MSCs (Figure 3E and 3F).

### Oxidant Stress and Nox4 Expression Are Greater in db/db Than in WT MSCs

**Figure 4.**
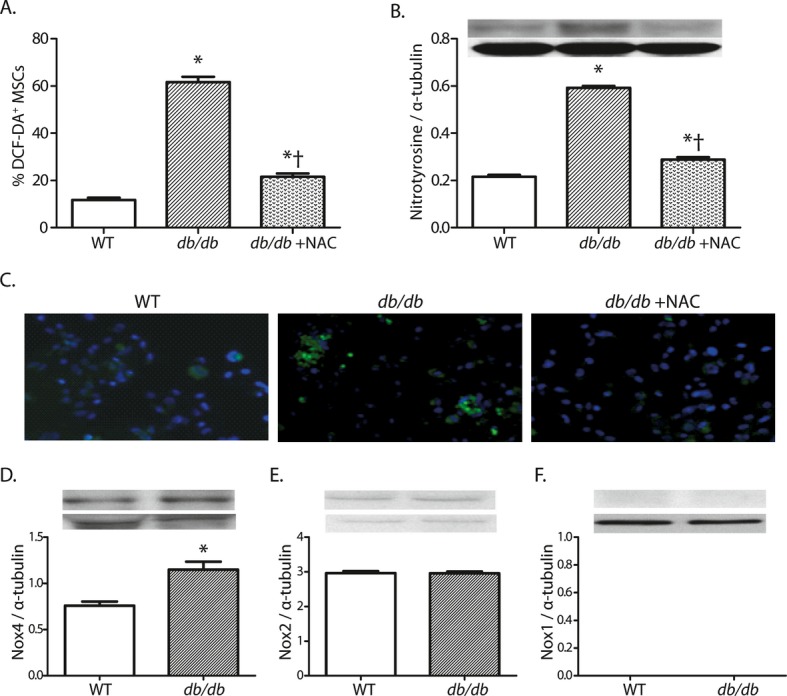
Oxidant stress and NADPH oxidase expression in *db/db* and WT MSCs. A and B, Oxidant levels, as determined by DCF staining and nitrotyrosine accumulation in MSCs (mean±SEM, n=6; *P<0.05 vs WT, †*P*<0.05 vs *db/db*). C, Representative photomicrographs of DCF staining in MSCs (×200). D through F, Quantitative expression of Nox4, Nox2, and Nox1 proteins in MSC cell lysates (D through E: mean±SEM, n=6; *P<0.05; F: n=6). WT indicates wild-type; MSCs, mesenchymal stem cells; and DCF, 2,7-dichlorofluorescein diacetate.

We next determined the presence of oxidant stress in MSCs. The percentage of MSCs that stained with 2,7-dichlorofluorescein diacetate (DCF) was >5-fold greater in those from *db/db* than in those from WT mice (Figure 4A and 4C). Nitrotyrosine expression in MSC cell lysates was 3-fold greater in *db/db* than in WT MSCs, indicating greater oxidant stress in *db/db* MSCs (Figure 4B). Treatment of *db/db* MSCs with the antioxidant *N*-acetylcysteine (NAC) significantly reduced the percentage of *db/db* MSCs stained with DCF and also reduced the expression of nitrotyrosine in these cells (Figure 4A through 4C).

Several isoforms of the NADPH oxidase family make major contributions to oxidant levels in bone marrow–derived stem cells,^17^ although specific expression of these isoforms in MSCs is unknown. The *db/db* MSCs exhibited 1.5-fold greater expression of Nox4 than did WT MSCs (Figure 4D), whereas cells from both groups expressed similar levels of Nox2 and neither group expressed measurable levels of Nox1 (Figure 4E and 4F). The difference in Nox4 expression may be of particular relevance to the increased adipogenic response of *db/db* MSCs because Nox4 expression can be induced by insulin^18^ and is essential for the differentiation of 3T3-L1 preadipocytes toward an adipocytic phenotype.^16,18^

### Multipotency of db/db MSCs Is Restricted in an Oxidant-Dependent Manner

**Figure 5.**
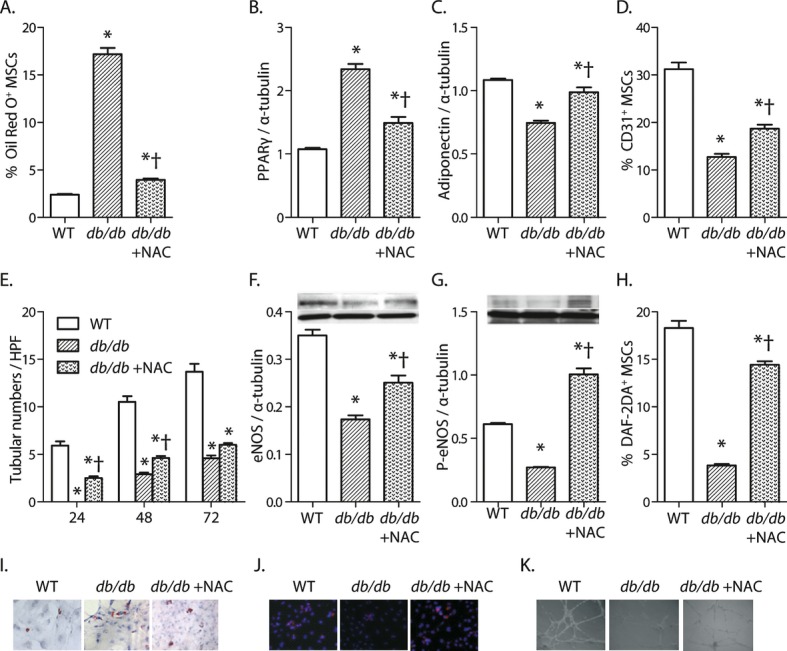
In vitro responses of MSCs to selective differentiation media. A, Quantification of MSC differentiation toward adipocytes. B and C, Quantification of PPARγ and adiponectin expression in MSCs. D, Quantification of MSC differentiation toward endothelial cells. E, Quantification of MSC tubular formation capacity. F through H, Quantification of eNOS and P-eNOS and NO levels in MSCs. (For A through H: mean±SEM, n=6; *P<0.05 vs WT, †*P*<0.05 vs *db/db*.) I, Representative photomicrographs of adipocyte differentiation (Oil Red O staining, hematoxylin counter stain, ×200). J, Representative photomicrographs of endothelial differentiation (CD31 immunostaining [red]; DAPI counter stain [blue], ×200). K, Representative photomicrographs of tubular structure from each group (×100 magnification). MSCs indicates mesenchymal stem cells; WT, wild-type; eNOS, endothelial NOS; P-eNOS, phosphorylated eNOS; and NO, nitric oxide.

**Figure 6.**
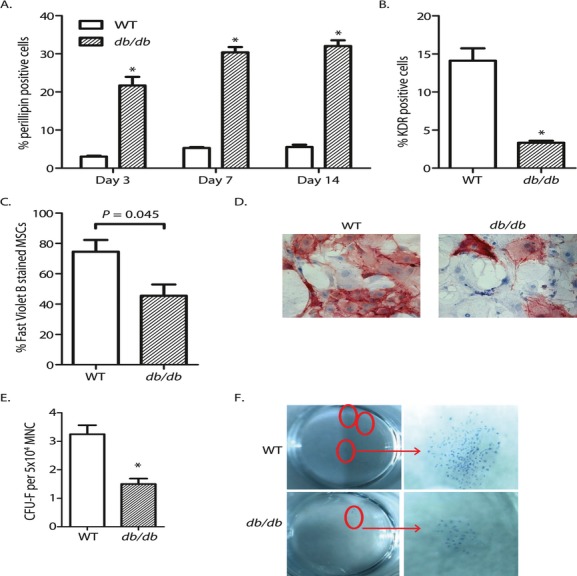
Differentiation and colony-forming unit assay of *db/db* and WT MSCs. A, *db/db* MSCs showed increased differentiation toward adipocytes by adipocyte marker perilipin staining (n=5). B, Fewer *db/db* MSCs differentiated into endothelial cells by staining of endothelial marker KDR (n=6). C, Fewer *db/db* than WT MSCs demonstrated differentiation to an osteocyte phenotype in response to osteogenic medium (n=5 to 6). D, Representative photomicrographs of WT and *db/db* MSCs following exposure to osteogenic differentiation medium (Fast violet B staining, hematoxylin counter stain, ×400). E, Significantly decreased colony-forming ability was observed in *db/db* MSCs (n=8). F, Representative colony images of WT and *db/db* MSCs following toluidine blue staining at high and low magnification of stereomicroscope. (For all panels: mean±SEM, *P<0.05.) WT indicates wild-type; MSCs, mesenchymal stem cells.

We next sought to clarify the molecular mechanisms for the restricted multipotency of *db/db* MSCs. Under adipogenic medium, 7-fold more *db/db* than WT MSCs differentiated into adipocytes, and this increased rate of differentiation of *db/db* MSCs into adipocytes was reversed by pretreatment with NAC (Figures 5A, 5I, and 6A).

Expression of PPARγ was greater, whereas expression of adiponectin was less in *db/db* than in WT MSCs. Treatment of *db/db* MSCs with NAC reversed these differences between groups (Figure 5B and 5C).

When 50 ng/mL VEGF and 2% FCS was added to the Mesencult culture medium, >3-fold more WT than *db/db* MSCs differentiated into endothelial cells (Figures 5D, 5J, and 6B). Pretreatment of *db/db* MSCs with NAC increased their rate of endothelial differentiation. Expression of endothelial NOS (eNOS) and phosphorylated eNOS (P-eNOS) and the percentage of nitric oxide (NO)–positive MSCs were greater in WT than in *db/db* MSCs (Figure 5F through 5H). Treatment of *db/db* MSCs with NAC increased their expression of eNOS and P-eNOS and their nitric oxide (NO) level. Finally, to determine whether type 2 diabetes affected MSC multipotency more broadly, we quantitated the capacity of MSCs to differentiate into other cell types, specifically osteocytes. More WT MSCs than *db/db* MSCs differentiated into an osteocyte phenotype under osteogenic medium (Figure 6C and 6D).

We next assessed the angiogenic capacity of MSCs in vitro. MSCs (1×10^5^) were seeded in 24-well plates that contained BD matrigel matrix. We counted the number of tubular structures 24, 48, and 72 hours later (Figure 5E and 5K). After 24 hours, no tubular structure formation was observed in *db/db* MSCs. NAC pretreatment of *db/db* MSCs increased the rate of tubular formation, although the rate remained lower than that in WT MSCs. In addition, WT MSCs showed significantly higher colony-forming capacity (Figure 6E and 6F).

Collectively, these in vitro findings corroborate the in vivo finding that the multipotency of *db/db* MSCs is restricted as their capacity for adipocyte differentiation is increased, whereas their capacity for endothelial cell and osteocyte differentiation is decreased. The reversal of this restriction by NAC indicates that MSC oxidant stress plays a central role in the restriction of MSC multipotency.

### Nox4 siRNA Reduces Nox4 Expression and Oxidant Levels in db/db MSCs and Restores MSC Multipotency

**Figure 7.**
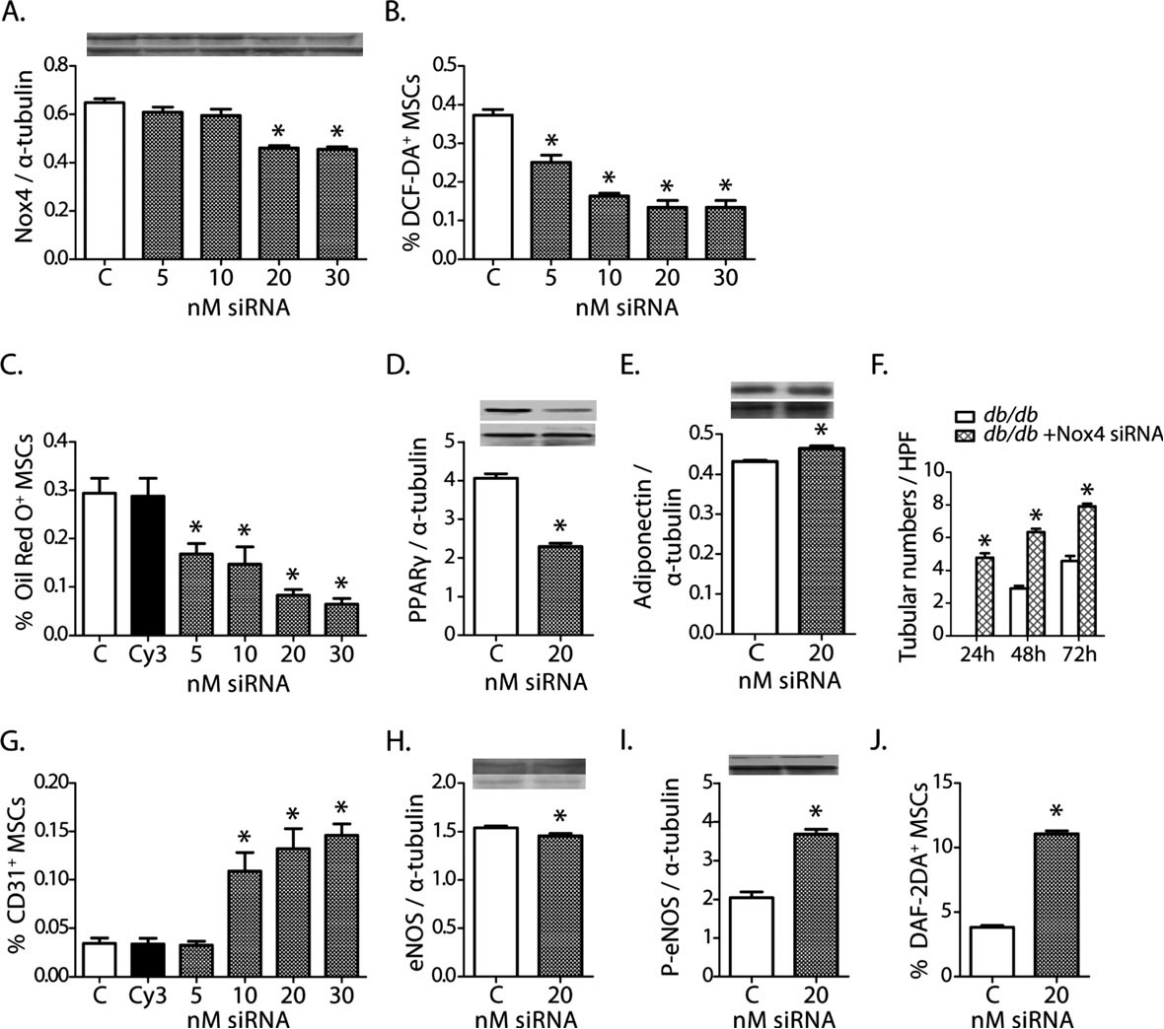
Effects of Nox4 siRNA on *db/db* MSCs. A, Nox4 siRNA reduced Nox4 expression in MSCs. B, Nox4 siRNA reduced oxidant levels in *db/db* MSCs (DCF staining). C, Nox4 siRNA reduced differentiation of *db/db* MSCs into an adipocyte phenotype. D and E, Nox4 siRNA reduced expression of PPARγ but had no effect on adiponectin expression. F, Nox4 siRNA increased *db/db* MSCs' tubular formation capacity. G, Nox4 siRNA increased differentiation of *db/db* MSCs into an endothelial phenotype. H through J, Nox4 siRNA increased P-eNOS expression and bioavailable NO, but had no effect on eNOS expression. (For all panels: mean±SEM, n=6; *P<0.05 vs control [C]; Cy3 is the siRNA control.) MSCs indicates mesenchymal stem cells; P-eNOS, phosphorylated eNOS; NO, nitric oxide; and eNOS, endothelial NOS.

We hypothesized that the increased Nox4 expression in *db/db* MSCs was central to the increased propensity for adipogenesis of these cells and was based on the established role of Nox4-derived H_2_O_2_ in terminal adipocyte differentiation.^16,18^ Nox4 siRNA was used to knock down Nox4 expression in *db/db* MSCs in vitro. Nox4 siRNA reduced Nox4 expression in *db/db* MSCs in a dose-dependent manner and reduced the percentage of *db/db* MSCs that stained with DCF, indicating that Nox4 is an important source of oxidant stress in these cells (Figure 7A and 7B). Pretreatment of *db/db* MSCs with Nox4 siRNA prior to their exposure to adipocyte differentiation medium reduced the percentage of cells that assumed an adipocyte phenotype (Figure 7C). Nox4 knockdown also reduced *db/db* MSC expression of PPARγ and increased expression of adiponectin (Figure 7D through 7E). Pretreatment of *db/db* MSCs with Nox4 siRNA prior to their exposure to endothelial differentiation medium increased the percentage of cells that differentiated into an endothelial phenotype (Figure 7G). Nox4 knockdown increased *db/db* MSC expression of phosphorylated eNOS and NO levels and slightly decreased eNOS expression (Figure 7H through 7J). Nox4 siRNA also increased the tubular formation of *db/db* MSCs (Figure 7F). The rate was greater than that in *db/db* MSCs treated with NAC, but remained lower than that of WT MSCs (Figures 5E, 5K, and 7F).

### Insulin Increases Nox4 Expression and Oxidant Levels of WT MSCs In Vitro and Thereby Restricts MSC Multipotency

**Figure 8.**
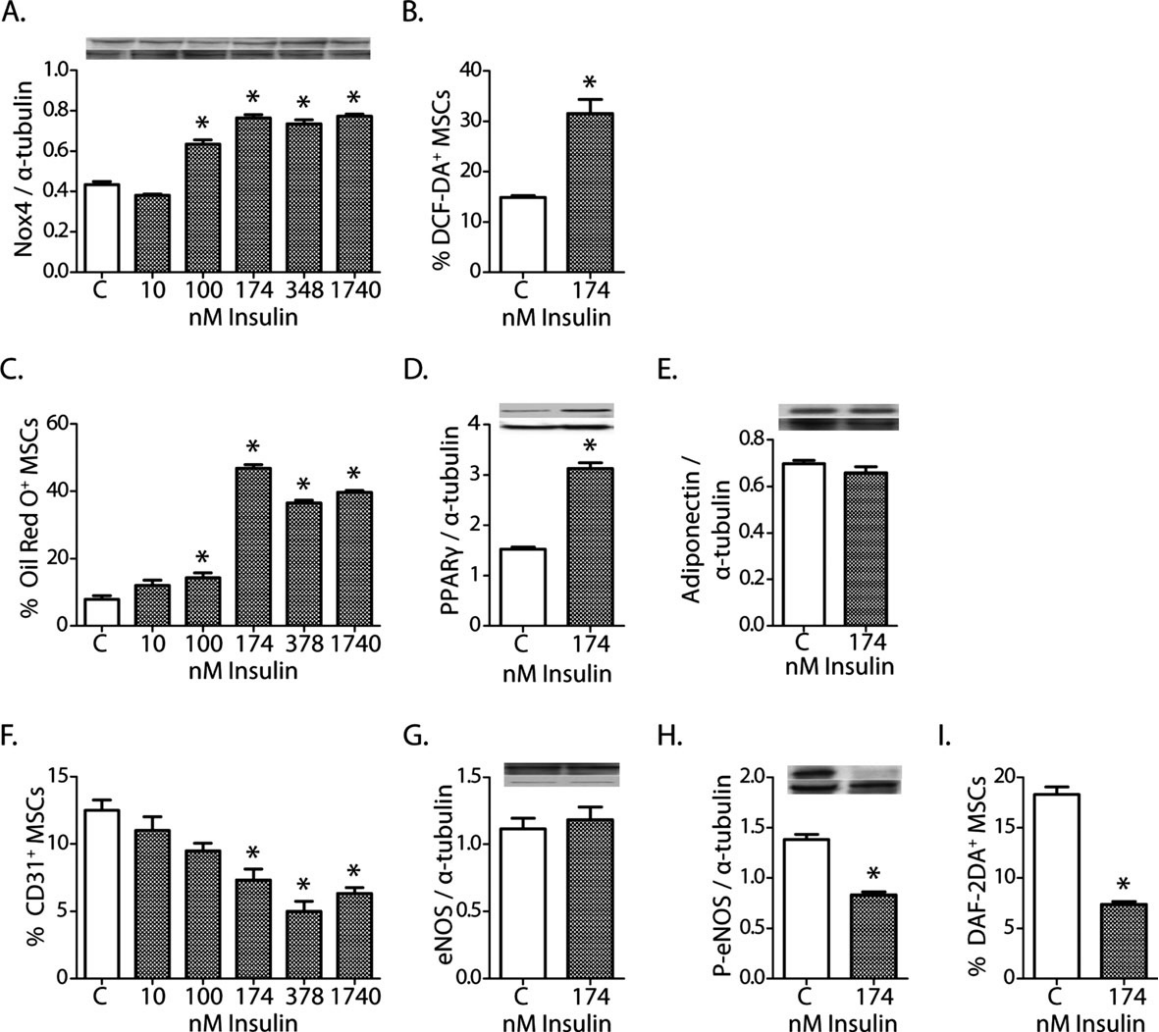
Effects of insulin on WT MSCs. A, Insulin increased Nox4 expression in WT MSCs. B, Insulin increased oxidant levels in WT MSCs. C, Insulin increased differentiation of WT MSCs into an adipocyte phenotype. D and E, Insulin increased expression of PPARγ but had no effect on adiponectin expression. F, Insulin decreased differentiation of WT MSCs into an endothelial phenotype. G through I, Insulin decreased P-eNOS expression and bioavailable NO but had no effect on eNOS expression. (For all panels: mean±SEM, n=6; *P<0.05 vs control [C]; Cy3 is the siRNA control. For all the Western blot images, up bands represent the expression of analyzed protein, and bottom bands represent the internal control, α-tubulin.) MSCs indicates mesenchymal stem cells; WT, wild-type; P-eNOS, phosphorylated eNOS; NO, nitric oxide; and eNOS, endothelial NOS.

**Figure 9.**
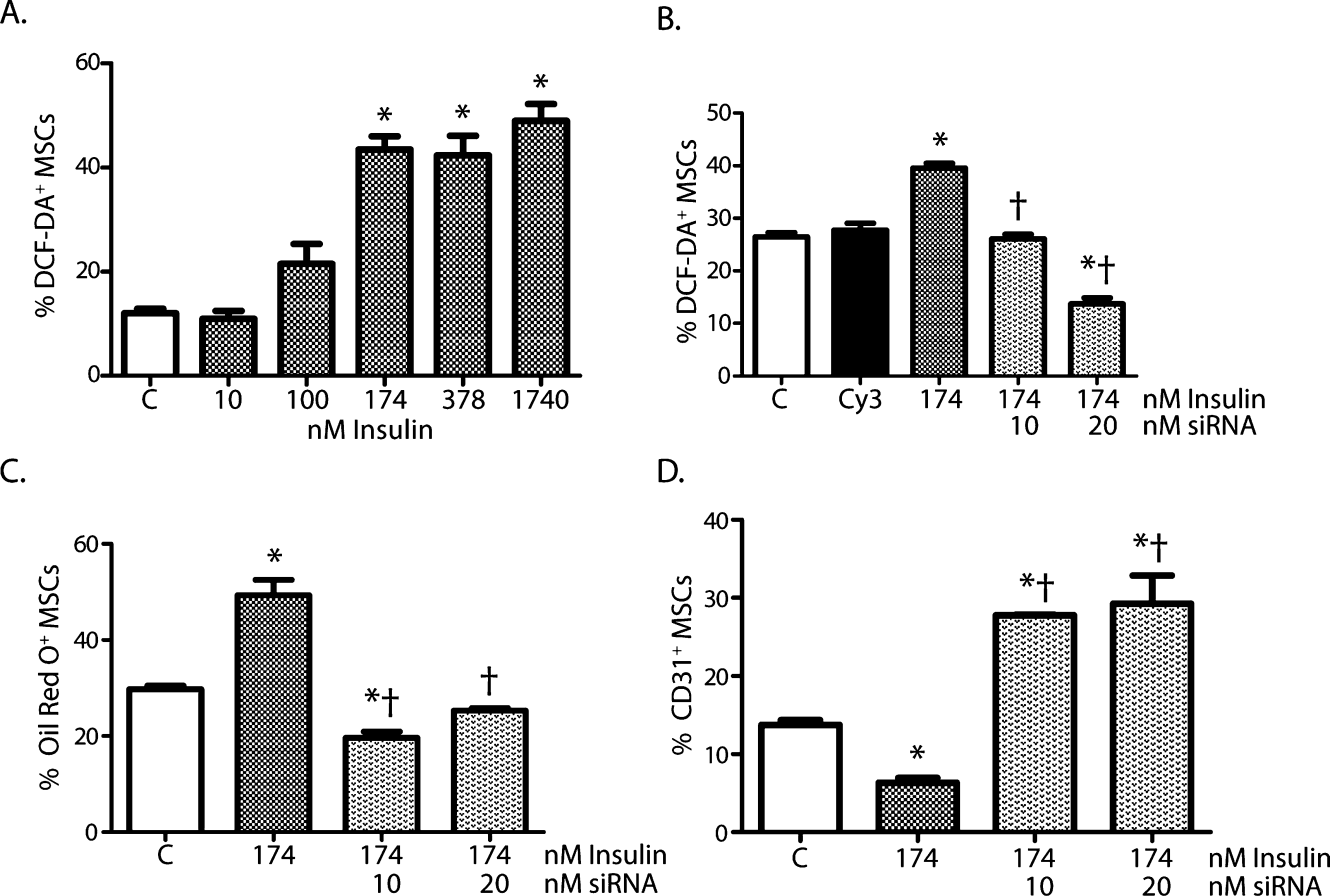
Effects of Nox4 on insulin-induced changes in WT MSCs. A, Insulin induced an increase in oxidant levels in WT MSCs. B, Pretreatment of WT MSCs with Nox4 siRNA blocked the pro-oxidant effects of insulin in WT MSCs. C, Pretreatment of WT MSCs with Nox4 siRNA blocked the effects of insulin on adipocyte differentiation. D, Pretreatment of WT MSCs with Nox4 siRNA blocked the effects of insulin on endothelial differentiation. (For all panels: mean±SEM, n=6; *P<0.05 vs control [C], †*P*<0.05 vs insulin alone; Cy3 was used as the siRNA control.) MSCs indicates mesenchymal stem cells; WT, wild-type.

Insulin is a critical participant in terminal adipocyte differentiation and acts, in part, by increasing Nox4 expression.^16,18^ We thus hypothesized that treatment of WT MSCs with insulin replicates the restricted differentiation capacity of *db/db* MSCs, that is, that insulin should increase Nox4 expression and oxidant stress, leading to restriction of MSC multipotency. The addition of insulin to basal medium increased Nox4 expression in WT MSCs in a dose-dependent manner and increased by 2-fold the percentage of WT MSCs that stained for DCF (Figures 8A, 8B, and 9A). Pretreatment of WT MSCs with insulin prior to their exposure to adipogenic medium increased the percentage of these cells that differentiated into an adipocyte phenotype. Insulin increased the expression of PPARγ in WT MSCs but had no effect on the expression of adiponectin (Figure 8C through 8E). Pretreatment of WT MSCs with insulin prior to their exposure to endothelial differentiation medium decreased the percentage of cells that differentiated into an endothelial phenotype. Insulin reduced expression of P-eNOS, and NO levels by WT MSCs, but did not affect expression of eNOS (Figure 8F through 8I).

**Figure 10.**
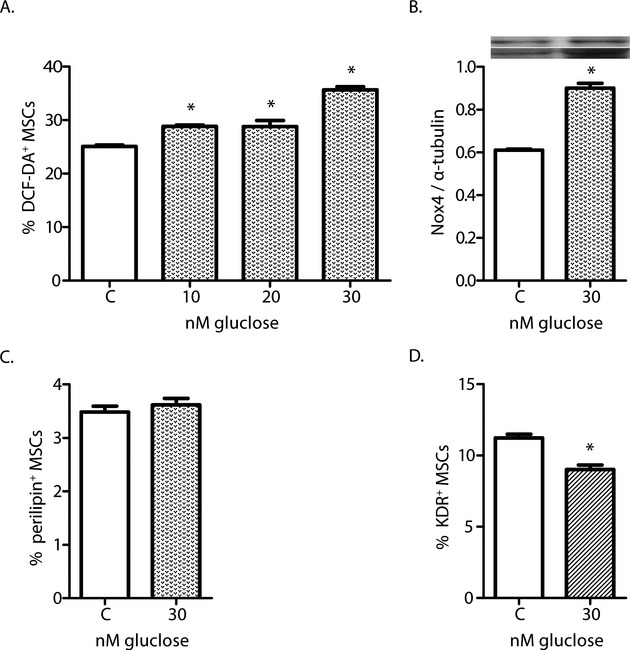
Effect of hyperglycemia on WT MSCs in vitro. A, Quantification of DCF staining of WT MSCs treated with 10, 20, or 30 nmol/L gluclose. B, Quantification of Nox4 expression in WT MSCs treated with 30 nmol/L glucose (vs control) by Western blot. C, Quantification of WT MSC differentiation toward adipocytes by FACS analysis of perilipin staining after treatment of MSCs with 30 nmol/L gluclose. D, Quantification of WT MSC differentiation toward endothelial cells by FACS analysis of KDR staining after treatment of MSCs with 30 nmol/L gluclose. (For all panels: (mean±SEM, n=5; *P<0.05 vs control.) MSCs indicates mesenchymal stem cells; WT, wild-type.

To determine if insulin resistance-induced hyperglycemia in *db/db* mice contributed to this restriction of MSC multipotency, we exposed WT MSCS to concentrations of glucose in vitro. Hyperglycemia increased oxidant stress and Nox4 expression in MSCs, but the oxidant stress increased to a lesser extent than that induced by hyperinsulinemia (Figures 8A, 9A, 10A, and 10B). However, the hyperglycemia-induced oxidant stress had no effect on the rate of differentiation of WT MSCs into adipocytes; but it did slightly decrease their rate of differentiation into endothelial cells. (Figure 10C and 10D). These results support hyperinsulinemia-induced Nox4 expression as the primary determinant of the restricted multipotency of MSCs in *db/db* mice.

### Nox4 siRNA Reverses Effects of Insulin-Induced Restriction of WT MSC Multipotency

We next sought to confirm that the changes in oxidant stress and differentiation capacity of insulin-treated WT MSCs were secondary to the insulin-induced increase in Nox4 expression. WT MSCs pretreated with Nox4 siRNA and then exposed to insulin had a lower percentage of cells that stained for DCF than did cells treated with insulin alone, indicating that Nox4 was an important source of oxidant stress in these cells (Figure 9A and 9B). Pretreatment of WT MSCs with Nox4 siRNA prior to insulin also reversed the insulin-induced restriction in MSC multipotency. The percentage of WT MSCs that differentiated into an adipocytic phenotype decreased, whereas the percentage of WT MSCs that differentiated into an endothelial phenotype increased if Nox4 knockdown was carried out before the administration of insulin (Figure 9C and 9D). Collectively, these findings indicate that Nox4-derived oxidants restrict *db/db* MSC multipotency, causing enhanced potential for adipocytic differentiation and reduced potential for endothelial differentiation.

### Nox4 siRNA Reverses Oxidant Stress in Type 2 Diabetic MSCs and Restores Their Capacity to Augment Postischemic Neovascularization in *db/db* Mice In Vivo

**Figure 11.**
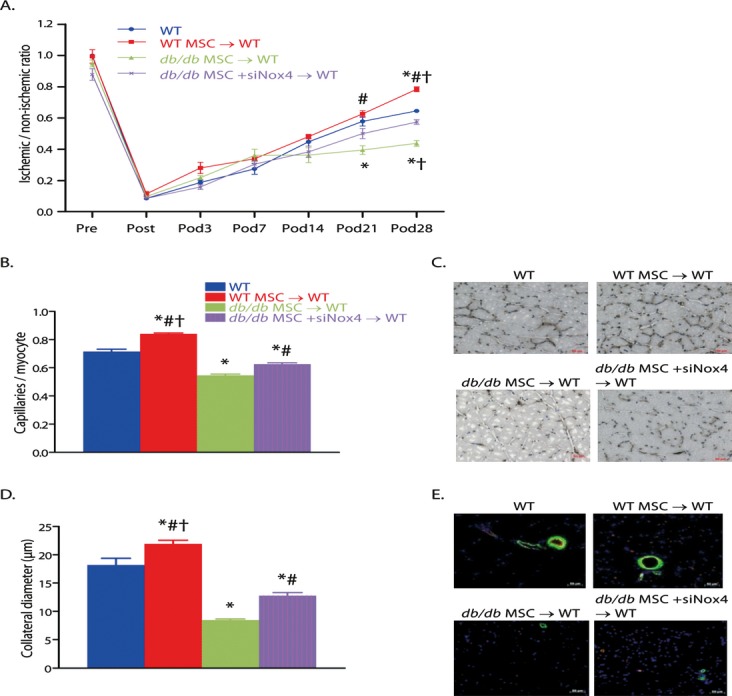
Reversal of Nox4-induced oxidant stress in type 2 diabetic MSCs restorefd their capacity to augment postischemic neovascularization in *db/db* mice. A, Foot blood flow recovery measurement by LDPI. B and C, Quantification (B) of capillary density by CD31 immunostaining and representative images (C). D and E, Quantification (D) of collateral diameter by CD31 and α-SMA double staining and representative images (E). (For all panels: mean±SEM, n=6; *P<0.05 vs WT, †*P*<0.05 vs *db/db* MSC+siNox4→WT transplant group, #*P*<0.05 vs *db/db* MSC→WT transplant group; scale bar, 50 μm.) WT indicates wild-type; MSCs indicates mesenchymal stem cells; and LDPI, laser Doppler perfusion imaging.

We transplanted Nox4 siRNA–transfected *db/db* MSCs into WT recipients 24 hours after induction of hind-limb ischemia. Blood flow recovery was measured at different times by laser Doppler scanning (Figure 11A). Recipients of *db/db* MSCs pretreated with Nox4 siRNA recovered blood flow to 57±2% that of the nonischemic limb, significantly higher than that of WT recipients of *db/db* MSCs. In addition, knockdown of Nox4 expression in *db/db* MSCs also increased mean collateral artery diameters and capillary density (Figure 11B through 11E). These results indicate that the impaired capacity of *db/db* MSCs to augment postischemic neovascularization in vivo is a result of Nox4-induced oxidant stress.

### Diet-Induced Diabetic Mice Also Showed Impaired Foot Blood Flow Recovery, Intramuscular Adipocyte Infiltration, and Impaired MSC Multipotency

*db/db* Mice exhibit hyperglycemia, hyperinsulinemia, obesity, and dislipidemia and have thus been widely used as an experimental model of type 2 diabetes mellitus.^11,19^ However, these mice are functionally leptin deficient, a feature that could affect studies of postischemic neovascularization insofar as leptin is a proangiogenic factor.^20^ We thus sought to confirm that observations made in *db/db* mice were consequent to their diabetic phenotype and not secondary to functional leptin deficiency or obesity. To this end, type 2 diabetes was induced in C57BL/6 mice by feeding the mice a high-fat diet.^13^ Diet-induced diabetic mice (DIDM) were not obese, and their fasting whole-blood glucose and plasma insulin levels were significantly greater than those of C57BL/6 mice. Importantly, however, whole-blood glucose and plasma insulin levels in DIDM were significantly lower than levels in *db/db* mice (Table 1).

**Table 1.** Body Weight, and Fasting Plasma Glucose and Insulin Levels in C57BL/6 Mice, *db/db* Mice, and DIDM

	Weight, g	Fasting Glucose, mg/dL	Fasting Insulin, ng/mL
C57BL/6	28±2	96±8	0.25±0.03

*db/db*	52±5*	324±46*	17.07±8.2*

DIDM	32±4†	195±10*	0.68±0.03*†

DIDM indicates diet-induced diabetic mice. Glucose was measured in whole blood, whereas insulin was measured in plasma.

* *P*<0.05 vs C57BL/6.

† *P*<0.05 vs *db/db*.

**Figure 12.**
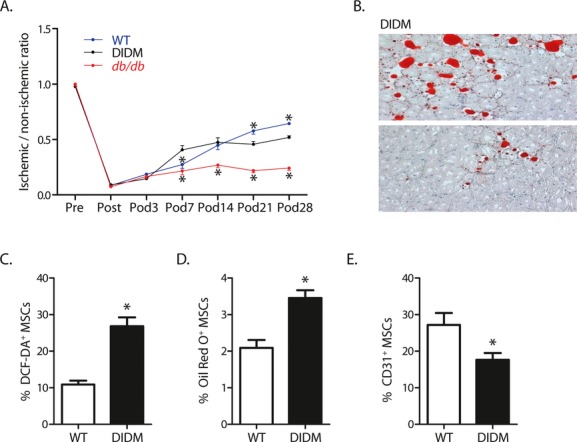
Diet-induced diabetes (DIDM) impaired recovery from hind-limb ischemia, induced adipocyte differentiation in ischemic muscle, and restricted MSC multipotency. A, Foot blood flow recovery in DIDM (mean±SEM, n=6; *P<0.05 vs DIDM; note that the WT data shown here were generated specifically for comparison with DIDM and are different from those displayed in Figure 3). B, Intramuscular adipocyte infiltration within ischemic hind-limb muscle from DIDM. C, Oxidant levels, as shown by DCF staining (n=7). D, MSCs from DIDM demonstrated increased differentiation to an adipocyte phenotype (n=8). E, MSCs from DIDM demonstrated reduced differentiation to an endothelial phenotype (n=7 to 8). (For C through E: mean±SEM; *P<0.05.) WT indicates wild-type; MSC indicates mesenchymal stem cell.

Recovery of foot blood flow in DIDM plateaued 2 weeks after induction of hind-limb ischemia, reaching 51.9±0.02% on POD28 (Figure 12A). We compared this recovery pattern to that we reported for C5lBL/6 and *db/db* mice. The percentage of foot blood flow recovery in DIDM was lower than that of C57BL/6 mice (64.5±0.01%), but higher than that of *db/db* mice (24.1±0.02%) on POD28 (both *P*<0.05 versus DIDM). DIDM showed adipocyte infiltration within the ischemic gastrocnemius muscle that was similar to that previously reported in *db/db* mice (Figure 12B).^4^ These findings confirm that the decreased postischemic foot blood flow recovery and the adipocytic infiltration of ischemic muscle were not solely consequent to the functional leptin deficiency present in *db/db* mice.

DIDM MSCs had a greater percentage of DCF-positive cells than WT MSCs (Figure 12C). Interestingly, the percentage of DCF positive MSCs in DIDM (26.8±2.45%) was significantly less than that observed in *db/db* mice (61.7±2.23% [Figure 4A], *P*<0.05 versus DIDM), a pattern that mirrored the quantitative differences in insulin in these groups. The percentage of DIDM MSCs that assumed an adipocyte phenotype in response to selective differentiation medium was greater than that of WT MSCs (Figure 12D); however, this percentage was less in DIDM (3.5±0.21%) than in *db/db* mice (19.6±0.16% [Figure 5A], *P*<0.05 versus DIDM). The percentage of DIDM MSCs that assumed an endothelial phenotype in response to selective differentiation medium was less than that in WT MSCs (Figure 12E); however, this percentage was greater in DIDM (17.6±1.89%) than in *db/db* MSCs (12.7±0.68% [Figure 5D], *P*<0.05 versus DIDM). These findings suggest that the effect of oxidant stress on MSC multipotency is likely dose dependent and confirm that the restriction of MSC multipotency in *db/db* mice is a result of the presence of type 2 diabetes and not of the functional leptin deficiency in *db/db* mice.

## Discussion

We report for the first time that experimental type 2 diabetes induces oxidant stress in MSCs, restricts their multipotency, and impairs MSC-induced augmentation of blood flow recovery after induction of hind-limb ischemia. In vivo differentiation of transplanted *db/db* MSCs into adipocytes was evident in the ischemic hind-limb muscle of WT mice. Crossno et al^21^ also reported adipocytic differentiation of transplanted MSCs, but only within existing adipose tissue; here, however, the differentiation of MSCs into adipocytes was most certainly pathological, as the adipocytes were present between myofibers of ischemic muscle, were accompanied by a concomitant decrease in the number of MSC-derived endothelial cells, and were present after transplantation of *db/db* MSCs, but not WT MSCs. In vitro studies showed that MSC oxidant stress was generated by an hyperinsulinemia-induced increase in Nox4 expression. The implications of the these findings are considerable, as they demonstrate a novel mechanism by which diabetes-induced oxidant stress compromises MSC multipotency and thus reduces their effective participation into postischemic neovascularization.

Transplanted *db/db* MSCs imposed 2 features of the *db/db* phenotype on WT recipient mice: impairment of postischemic foot blood flow recovery and intramuscular adipocyte infiltration. One reason for the deleterious effect of *db/db* MSCs on postischemic blood flow recovery is the restriction of MSC multipotency. Colocalization studies confirmed that a substantial percentage of transplanted *db/db* MSCs that homed in on the ischemic hind limb assumed an adipocytic phenotype, whereas very few of the transplanted cells differentiated into an endothelial cell phenotype. MSCs can exert their beneficial effects in vascular repair by 2 means: first, by engraftment into blood vessel walls and differentiation into endothelial cells, pericytes, or other vascular cell phenotypes^5,22,23^; and second, by paracrine mechanisms, including generation of vascular growth factors and proangiogenic cytokines.^6,24^ MSCs that have differentiated into an adipocytic phenotype would be improbable candidates for subsequent transdifferentiation into an endothelial cell phenotype. Adipocyte differentiation of *db/db* MSCs could therefore negate any contribution these cells might make to postischemic neovascularization.

The present findings establish that hyperinsulinemia-induced, Nox4-generated oxidant stress is the mechanistic basis for the restricted multipotency and increased adipogenic predilection of *db/db* MSCs. Two lines of evidence support this conclusion. First, NAC or Nox4 siRNA knockdown reduced the oxidant stress in *db/db* MSCs and decreased their rate of differentiation into adipocytes. And, second, insulin treatment of WT MSCs increased Nox4 expression, increased oxidant stress, and increased the rate of differentiation into adipocytes. Moreover, these insulin-induced effects were reversed by Nox4 knockdown prior to insulin treatment, confirming the pivotal role of Nox4 in this response. Mahadev et al^18^ and Schroder et al^16^ have reported that insulin-induced Nox4 participates in terminal differentiation of 3T3-L1 preadipocytes to adipocytes. Indeed, insulin-induced Nox4 expression has been proposed as the key “switch” in the signal transduction pathway that directs preadipocytes to undergo terminal differentiation, rather than to continue proliferation as preadipocytes.^16^ This concept is entirely consistent with our present findings, which have demonstrated that Nox4-generated oxidant stress directs the decision of the MSC lineage fate toward adipocyte differentiation and away from endothelial cell differentiation. MSC multipotency is sensitive to oxidant stress.

Although the in vitro findings conclusively implicate insulin-induced, Nox4-generated oxidants as pivotal in MSC adipogenesis, gaps in understanding of this mechanism remain. For example, we identified changes in the MSC expression of the nuclear receptor PPARγ that were consistent with earlier reports that indicated PPARγ is both requisite and sufficient to induce terminal adipocyte differentiation^25^ and that oxidants amplify PPARγ expression.^26^ Similarly, we determined that MSC expression of activated (phosphorylated) eNOS and NO were increased by Nox4 knockdown and reduced by insulin, both in an oxidant-dependent manner. eNOS-derived NO is a principal stimulus for endothelial cell differentiation.^27–29^ Findings made in diet-induced diabetic mice (DIDM) confirm that the aberrations in postischemic neovascularization and MSC function observed in *db/db* mice were not the singular consequence of the functional leptin deficiency that characterizes the *Lepr*^*db/db*^ genotype,^19^ insofar as qualitatively similar observations were made in the DIDM group. However, the metabolic phenotype generated in the DIDM group was dissimilar to *db/db* mice in one crucial respect: the fasting plasma insulin in DIDM was much lower than that observed in *db/db* mice. We believe that this difference in fasting plasma insulin underlies the lesser degree of MSC oxidant stress observed in DIDM that, in turn, is responsible for the more limited response of DIDM-derived MSCs than *db/db* MSCs to adipogenic differentiation medium. This supposition is based on the pivotal role of hyperinsulinemia-induced, Nox4-derived oxidants in enhancing the adipogenic potential of MSCs established by the in vitro findings of this study. DIDM exhibited foot blood flow recovery that was significantly less than that of C57BL/6 mice but was not as severely deficient as that of *db/db* mice, and it is more convincing that the moderate changes in MSC function that occurred in this group were responsible, at least in part, for the more limited compromise in postischemic neovascularization in these mice than that seen in *db/db* mice. Leptin is a key adipokine.^30^ However, leptin does not affect terminal differentiation of preadipocytes.^30,31^ The adipocytic infiltration within the ischemic muscle in DIDM confirms that factors other than leptin deficiency are responsible for this effect.

MSCs have been proposed as a therapeutic option for a wide variety of diseases, including peripheral arterial occlusive disease, on the basis of their ease of harvest, rapid *ex vivo* proliferation, and established efficacy in improving vascular recovery in preclinical models of ischemia.^5,7,8^ Some evidence suggests that MSCs exert an immunosuppressive effect,^32^ whereas other work indicates that these cells can become immunogenic,^33^ leaving an uncertainty that makes autologous MSC transplantation most feasible at present. MSCs harvested from diabetic mice demonstrate oxidant-dependent dysfunction; thus, instead of increasing blood flow recovery in the ischemic limb, they reduce it, because cells that reach ischemic tissues have restricted multipotency and differentiate into adipocytes. However, in vitro observations indicate that antioxidant treatment restores redox balance and differentiation capacity in MSCs from diabetic mice. Moreover, we confirmed in an in vivo model that pretreatment of *db/db* MSCs with Nox4 siRNA significantly increased their capacity to augment the neovascularization capacity of *db/db* mice to that of WT mice. The present findings might permit greater leverage of the therapeutic potential of autologous MSC transplantation in the setting of diabetes or any clinical condition that increases systemic oxidant stress, as do all the risk factors for cardiovascular disease. Thus, in vivo systemic treatment or *ex vivo* treatment of MSCs with antioxidants or both might significantly improve the posttransplantation efficacy of these cells in exerting the intended clinical benefit and thereby realize the full promise of MSC therapy.
